# Data on genome resequencing of pigmented and non-pigmented Malaysian rice varieties

**DOI:** 10.1016/j.dib.2020.105806

**Published:** 2020-06-03

**Authors:** Rabiatul-Adawiah Zainal-Abidin, Zamri Zainal, Zeti-Azura Mohamed-Hussein, Yun Shin Sew, Sanimah Simoh, Shahril Ab Razak, Norliza Abu-Bakar

**Affiliations:** a Biotechnology and Nanotechnology Research Centre, Malaysian Agricultural Research & Development Institute (MARDI), 43400 Serdang, Selangor, Malaysia; b Institute of Systems Biology (INBIOSIS), Universiti Kebangsaan Malaysia, 43600 UKM Bangi, Selangor, Malaysia; c Centre for Frontier Sciences, Faculty of Science & Technology, Universiti Kebangsaan Malaysia, 43600 UKM Bangi, Selangor, Malaysia

**Keywords:** Genome, HiSeq, Nutritional, Pigmented rice, Single nucleotide polymorphism (SNP)

## Abstract

The genomics and genetic data of pigmented and non-pigmented Malaysian rice varieties are still limited. Hence, we performed the genome resequencing of two black rice varieties (Bali, Pulut Hitam 9), two red rice varieties (MRM16, MRQ100) and two white rice varieties (MR297 and MRQ76) using Illumina HiSeq 4000 platform with 30x sequencing coverage. We aimed to identify and annotate single nucleotide polymorphisms (SNPs) from the genome of these four pigmented and two non-pigmented rice varieties. The potential SNPs will be used in developing the functional SNP markers related to nutritional (i.e. antioxidant, folate, amylose) and quality (i.e. aromatic) traits. Raw data of the pigmented and non-pigmented rice varieties have been deposited into the European Nucleotide Archive (ENA) database with accession number PRJEB29070 and PRJEB32344, respectively.

Specifications Table

**Table:** 

Subject	Agricultural and Biological Sciences
Specific subject area	Plant sciences
Type of data	Genome sequence data
How data were acquired	Illumina HiSeq 4000 sequencing platform
Data format	Clean raw reads (FASTQ)
Parameters for data collection	Total DNA was extracted from leaves of two weeks old germinated seedlings using Murray and Motou protocols and Sigma DNA extraction kit.
Description of data collection	The leaves of two-week-old germinated seedlings were obtained from each pigmented and non-pigmented Malaysian rice varieties and quickly frozen in liquid nitrogen before stored in a −80C freezer.
Data source location	City/Town/Region: Serdang, Selangor Country: Malaysia Latitude and longitude (and GPS coordinates) for collected samples/data:] 2.9885871″N 101.697955417″E
Data accessibility	The raw reads of pigmented and non-pigmented rice varieties have been deposited at European Nucleotide Archive (ENA) (https://www.ebi.ac.uk/ena) under the studies accession numbers PRJEB29070 (pigmented) and PRJEB32344 (non-pigmented). https://www.ebi.ac.uk/ena/data/view/PRJEB29070 https://www.ebi.ac.uk/ena/data/view/PRJEB32344
Related research article	Zainal-Abidin, R., Abu-Bakar, N., Sew, Y., Simoh, S & Mohamed-Hussein, Z-A, Discovery of functional SNPs via Genome-Wide Exploration of Malaysian Pigmented Rice Varieties. 2019. International Journal of Genomics. doi.org/10.1155/2019/4,168,045.

Value of the Data

• The pigmented and non-pigmented genome data provide genomics and genetics data of Malaysian rice varieties, which can be utilized in investigating their genetic basis.
• Molecular markers such as single nucleotide polymorphisms (SNPs) and microsatellites can be discovered from the genomes and will be useful in facilitating genetic improvement of rice varieties in order to enhance their nutritional and quality traits.
• These genome data are valuable for evolutionary study and the identification of domestication genes.

## 1 Data

The genomes of four Malaysian pigmented and two non-pigmented rice varieties were sequenced using Illumina HiSeq 4000 sequencing technology, with the read length of 150 bp at each end. We used a 30x depth of sequencing coverage. Approximately 88 Gb raw data sequenced were generated from the genomes of four Malaysian pigmented and two non-pigmented rice varieties. After filtering the low-quality data, we obtained 87 Gb clean reads and these clean reads were used for further analyses, such as reads mapping and SNPs calling. Table 1 shows the summary of raw and clean reads that have been generated from the genome resequencing of pigmented and non-pigmented rice varieties. The clean raw reads of pigmented and non-pigmented rice varieties have been deposited at European Nucleotide Archive (ENA) (https://www.ebi.ac.uk/ena) under the studies accession numbers PRJEB29070 (pigmented) and PRJEB32344 (non-pigmented).

**Table 1:** Summary of raw data for each pigmented and non-pigmented rice varieties.

Varieties	Phenotype	Total raw nucleotides (bp)	Total clean nucleotides (bp)	ENA Studies Primary Accession	ENA Run Primary Accession
Bali	Pigmented (Black)	15,256,585,800	14,979,784,200	PRJEB29070	ERR2831548
Pulut Hitam 9	Pigmented (Black)	14,997,625,200	14,907,066,900	PRJEB29070	ERR2831549
MRM16	Pigmented (Red)	14,814,608,700	14,711,718,300	PRJEB29070	ERR2831551
MRQ100	Pigmented(Red)	14,245,605,300	14,164,294,800	PRJEB29070	ERR2831550
MR297	Non-pigmented (White)	14,525,856,000	14,418,249,900	PRJEB32344	ERR3299466
MRQ76	Non-pigmented (White)	14,480,258,100	14,387,893,500	PRJEB32344	ERR3299467
Total		88,320,539,100 ∼88.32 Gb	87,569,007,600 ∼87.57 Gb		

## 2 Experimental design, materials, and methods

### 2.1 Sample preparation and sequencing

The genomic DNA was extracted from the young leaves of each pigmented and non-pigmented rice varieties using Murray [1] and Motou [2] protocols and Sigma DNA extraction kit. NanoDrop spectrophotometer (Thermo Scientific, Waltham, MA, USA) was used to check the DNA quality and quantity. The 0.8% agarose gel was used to examine the integrity of DNA samples. The DNA samples were sent to the sequencing service provider at the Novogene Bioinformatics Technology Co., Ltd. (Beijing, China). Paired-end sequencing libraries with insert sizes of 350 bp were constructed according to the manufacturer's standard protocol (Illumina, San Diego, CA, USA). Paired-end sequencing was performed using Illumina HiSeq 4000 sequencing platform with the read length of 150 bp at each end.

## Declaration of Competing Interest

The authors declare that they have no known competing financial interests or personal relationships that could have appeared to influence the work reported in this paper.

## Appendix Supplementary materials

Image, application 1
